# The History and Capabilities of Herbal Simples

**Published:** 1890-09-13

**Authors:** W. T. Fernie


### The History and Capabilities of Herbal Simples.

By W. T. Fernie, M.D.

*(Continued from page 246.)*

### XVII.—CHICKWEED AND GROUNDSEL.

Chickweed, called Alsine, or *Stellaria media*, a floral star of middle magnitude, and belonging to the pink order of plants, is a most hardy and widely diffused annual weed, which fills every neglected corner of our gardens, and stands the severest winters. Together with the groundsel, it grows wild all over the earth, and serves as food for finches, linnets, and other small feathered songsters of the woods. Furthermore, *Qui alunt aviculas caveis inclusas, hoc solent illas, si quando cibos fastigiant, recreare*, or, as Gerard puts it, "Little birds in cages are refreshed with chickweed when they loath their meat." The steady persistence and hardihood of these two plants from earliest times, throughout Europe, North America, and Siberia, seem to show that they must be endowed with some universal uses, which have enabled them to hold their own until now in the great evolutionary struggle. For these reasons I propose to consider them together. Moreover, the fact of our still finding chickweed and groundsel in England as well as on the mainland of Europe affords another proof that when Britain was repeopled after the great ice-age it must have been united somewhere to the Continent. The chickweed is termed alsine—*quia lucos, vel alseous amat*—because it loves to grow in shady places. Both it and the groundsel abound with the earthy salts of potash, which are admirable against scurvy when thus found in nature's laboratory; and a continued deprivation from which always proves disastrous to mankind, as was seen in our Arctic explorers when left without green vegetables or limejuice. "The water of chickweed," says an old writer, "is given to children for their fits, and its juice is used for their gripes." A poultice prepared from the fresh, green, juicy leaves is emollient and cooling; and an ointment of them made with hog's lard is healing. In the language of flowers the plant is interpreted as "rendezvous." The herb is distinguished by its small starlike flowers, and by a line of diminutive stiff hairs which runs up one side of its stem, like a vegetable hogmane, and, when reaching a pair of leaves, immediately crosses over, and parts its hair on the other side. The plant is a capital indicator of weather, because, as Lord Bacon first observed, when rain is impending its flowers remain closed; whereas, when they are fully open, the umbrella may be safely left at home. Also the chickweed teaches a gentle matrimonial lesson, seeing that at night its leaves approach one another in loving pairs, and sleep with the tender buds of the plant protected between them. Culpeper says: "Chickweed is a fine, soft, pleasing herb, under the dominion of the moon, and good for many things." The leaves, boiled in vinegar and salt, are of use against manginess of the hands and legs, if bathed therewith. Its juice, if rubbed on warts, will cause them to fall away and disappear. Chickweed, when boiled, exactly resembles spinach. Probably Mr. Froggy, of the well-known nursery rhyme, took some of this green stuff, with a small joint of pork, as a present to Miss Mouse, his ladylove, when a-wooing he would go, whether his mother would let him or no. So "off he set, with his opera hat. When he came to the door he gave a loud rap, with his roley, poley, *gammon* and *spinach*. Heigho! said Anthony Roley."

In the hands of the simplers, groundsel formerly held high rank as a herb of power. An old herbal gives the following quaint use of the plant for toothache: "Dig up groundsel with a tool that hath no iron in it; touch the tooth five times with the plant, spit thrice after each touch, and the cure will then be complete." Hill tells us that the fresh roots, if smelled when first taken out of the ground, are an immediate cure for many forms of headache. Culpeper speaks of it as a gallant and universal medicine. "Lay by your learned recipes," he exclaims; "this herb alone, preserved in syrup, or in distilled water, or as an ointment, shall do the deed for you in all hot diseases, and shall do it first safely, then speedily." In an old manuscript of the fifteenth century, this plant is called grondeswyle, from grund, ground; and swelgan, swallow. To this day in Scotland it is named grundy-swallow, or ground-glutton. It belongs to the daisy tribe of herb, but has no white rays to its small yellow flower-heads. When these heads fall away, the grey woolly pappus of the seeds which is left collected in small bundles of silvery filaments, which are like the hoary hairs of age. They have suggested the name of the genus, senecio, to which the groundsel belongs. This is from the Latin, senex, an old man—"quod canis simili videatur flore capillis"—"With venerable locks the groundsel grows." And well may the same distich conclude thus: "Cura facit canum, quamvis vir non habet annos"— "Hard care more quick than years white headgear brings."

In the eastern counties of England the groundsel is named simson—pronounced as senshon. It flowers all the year round, but is not visited by insects, though it retains honey glands. Nevertheless, it is wind fertilised. The leaves are fleshy, with a bitter, saline taste; the juice is slightly acrid, but emollient. In this country farriers give it to horses for bott worms; and in Germany it is employed as a vermifuge [for children. A useful gargle is made from the groundsel in France for sore throats; two drachms by weight of the fresh plant being boiled in four fluid ounces of water. The same decoction when swallowed will act easily as a simple purge. It is efficacious outwardly for all kinds of sores, and for healing chapped hands. Our Folk Lore Society relates an old instance of a labourer's wife who, when suffering from ague, consulted a charmer, and was advised to bid her husband tie a handful of common groundsel and lay it in her bosom while the charmer said certain incantations. Parkinson, in his "Botanic Theatre," orders thus: "To make a salve fit to heal sore legs, boil a handful of chickweed, with a handful of red rose leaves, in a pint of the oil of trotters, or sheep's feet, and anoint the grieved places therewith, against a fire, each evening and morning; then bind some of the herb, if ye will, to the sore; and so shall ye find help, if God will." It is said that the bruised leaves will prevent boils; and the plant, if taken as a sallet, with vinegar, is good for sadness of the heart.

*(To be continued.)*

